# Fetal sex impacts birth to placental weight ratio and umbilical cord oxygen values with implications for regulatory mechanisms

**DOI:** 10.1186/s13293-022-00445-z

**Published:** 2022-06-29

**Authors:** Bryan S. Richardson, Akasham Rajagopaul, Barbra de Vrijer, Genevieve Eastabrook, Timothy R. H. Regnault

**Affiliations:** 1 Department of Obstetrics and Gynecology, Western University, London, Canada; 2 Department of Physiology and Pharmacology, Western University, London, Canada; 3 Department of Pediatrics, Western University, London, Canada; 4 Children’s Health Research Institute, London, Canada; 5 Department of Obstetrics and Gynecology, London Health Sciences Centre, Victoria Hospital, 800 Commissioners Road E, London, ON N6A 5W9 Canada

**Keywords:** Fetal sex, Placenta, Fetal oxygen, Fetal growth

## Abstract

**Background:**

We determined the effect of fetal sex on birth/placental weight and umbilical vein and artery oxygen values with implications for placental efficiency and regulatory mechanisms underlying fetal–placental growth differences.

**Methods:**

A hospital database was used to obtain birth/placental weight, cord PO_2_ and other information on patients delivering between Jan 1, 1990 and Jun 15, 2011 with GA > 34 weeks (*N* = 69,836). Oxygen saturation was calculated from the cord PO_2_ and pH data, while fractional O_2_ extraction was calculated from the oxygen saturation data. The effect of fetal sex on birth/placental weight, cord PO_2_, O_2_ saturation, and fractional O_2_ extraction was examined in all patients adjusting for pregnancy and labor/delivery covariates, and in a subset of low-risk patients.

**Results:**

Birth/placental weights were lower in females indicating decreased placental efficiency. Umbilical vein oxygen values were higher in females attributed to increased uterine blood flow, while artery oxygen values were lower in females attributed to decreased hemoglobin and umbilical blood flow, and increased oxygen consumption. Fetal O_2_ extraction was increased in females confirming increased O_2_ consumption relative to delivery.

**Conclusions:**

Sex-related differences in uterine/umbilical blood flows, placental development, and fetal O_2_ consumption can be linked to the differences observed in cord oxygen. The lower umbilical artery oxygen in females as a measure of systemic oxygenation signaling growth could account for their decreased birth weights, while slower development in female placentae could account for their lower placental weights, which could be differentially effected contributing to their lower birth/placental weights.

## Highlights

- Birth/placental wt is decreased in females as a measure of placental efficiency.
- Cord vein O_2_ is increased in females as a measure of placental O_2_ transport.
- Cord artery O_2_ is decreased in females as a measure of fetal systemic O_2_ levels.
- Sex differences in placental development link to cord O_2_-birth/placental wt findings.

## Background

There is considerable evidence that the placenta regulates nutrient delivery and consumption by morphological and functional adaptations to optimize fetal growth in response to maternal and fetal nutritional cues [1–3]. Accordingly, fetal weight for a given placental weight at birth is often used as a measure of ‘placental efficiency’ commonly defined as the grams of fetus produced per gram of placenta and has been studied in relation to varying pregnancy conditions [1, 3, 4]. This includes study in relation to fetal sex, where females are found to have lower birth weights, placental weights, and birth/placental weight ratios compared to males [4–7]. The decrease in birth weights relative to placental weights in females has been postulated to reflect decreased placental efficiency, but thereby with more reserve capacity and lessening vulnerability to nutritional deprivation [5]. While underlying mechanisms are unclear, this sex-related difference in birth/placental weight ratios could reflect differences in placental nutrient delivery and/or in growth-signaling processes and thereby nutrient consumption as seen during early embryonic development [6, 8, 9].

We have shown that umbilical vein and artery oxygen values relate to size at birth across the range of birth weights as studied at term supporting oxygen as a primary determinant of fetal growth in humans [10]. Umbilical vein oxygen values are directly related to utero-placental blood flow and the diffusion properties of the placenta which determine the transport capacity for oxygen and can be seen as a functional measure of placental O_2_ delivery [11–13]. Umbilical artery oxygen values are directly related to umbilical vein values and additionally umbilical blood flow which determine fetal O_2_ delivery [11–13]. Concurrently, umbilical artery values are inversely related to fetal O_2_ consumption and thereby fetal growth as a major component of oxidative metabolism [11–13]. It is the relationship of O_2_ delivery to the fetus and the rate of fetal O_2_ consumption which ultimately determines fetal systemic blood and tissue oxygen levels. To our knowledge umbilical vein and artery oxygen values have not been studied in relation to fetal sex, despite sex-related differences in birth/placental weight ratios as noted, and in uterine and umbilical blood flows [14–18], placental development [19, 20], and newborn O_2_ consumption [21], with the likelihood that cord oxygen values will also be differentially affected.

In the present study we determined the impact of fetal sex on birth/placental weight ratios as measures of differential growth and thereby placental efficiency, and umbilical vein and artery PO_2_ and O_2_ saturation as measures of placental O_2_ delivery and fetal systemic oxygen levels, respectively. We hypothesized that these outcome variables will show sex-related differences that can be attributed to predicted differences in placental and fetal O_2_ delivery and in fetal O_2_ consumption, providing insight for regulatory mechanisms underlying fetal–placental growth differences. Fetal fractional O_2_ extraction has additionally been studied as a measure of oxygen consumption relative to delivery and thereby of oxygen reserve available to the fetus [22], since sex-related differences could have clinical implications for the increased hypoxic-related morbidity/mortality seen in males [23, 24].

## Methods

The perinatal database of St Joseph’s Health Care, London, Canada, provides targeted information on all births that occurred at the hospital, with data prospectively entered by dedicated database personnel. The study population was formed based on the following inclusion criteria: all patients delivering between January 1, 1990, and June 15, 2011, when the delivery unit was closed and moved to London Health Sciences Centre; gestational age at birth greater than 34 completed weeks; singleton, live-born and no major anomalies, and known fetal sex at birth and thereby entered into the database. The database was used to obtain the following information for this study population: fetal sex as the primary independent variable; birth weight, placental weight, and umbilical vein and artery gases/pH, as primary outcome variables; and maternal age, parity, pre-pregnant body mass index (BMI), smoking status, pregnancy complications, gestational age at delivery, anesthetic use and delivery type, and non-reassuring fetal heart rate (FHR) pattern as indication for cesarean delivery as population characteristics and potentially confounding covariates. The study was approved by the Western University Human Subjects Research Ethics Board (no. 112567).

Consistent with clinical practice, gestational age was derived from the last menstrual period or corrected based on ultrasonography measurements [10]. An electronic weight scale was used by nursing personnel to weigh infants immediately after delivery. Placentaes were weighed with membranes and umbilical cord by nursing assistants, again using an electronic weight scale. Umbilical vein and artery blood were routinely sampled by nursing personnel immediately after delivery for all infants deemed to be viable [10].

Maternal pre-pregnant weight and height for determining BMI values were those reported by patients at their first prenatal visit. Fetal size at birth was divided into the following birth weight categories based on birth weight percentile in relation to weeks of pregnancy attained at the time of delivery and using the sex-specific neonatal growth nomograms of Kramer et al. [25]: (1) small for gestational age (SGA), birth weight < 10th percentile, (2) appropriate for gestational age (AGA), birth weight ≥ 10th percentile and ≤ 90th percentile, and (3) large for gestational age (LGA), birth weight > 90th percentile. Maternal smoking was scored as being present with any sustained use after pregnancy was diagnosed. Maternal pregnancy complications included chronic hypertension, pregnancy-induced hypertension, gestational diabetes, overt diabetes, and pre-term premature rupture of membranes using standard clinical criteria for these. Near term deliveries were those from 35 0/7 to 36 6/7 weeks, while post-term deliveries were those ≥ 41 weeks.

Cases that met the inclusion criteria were divided into two patient populations: (1) all patients, and (2) low-risk patients, a group that excluded patients who smoked, had chronic or pregnancy-induced hypertension, gestational or overt diabetes, SGA or LGA infants, delivery < 37 weeks, or delivery by cesarean section. Each patient population was studied independently and divided into two groups based on fetal sex as male or female.

### Data acquisition, calculations, and statistical analysis

Placental weights < 0.5th percentile and > 99.5th percentile were excluded to avoid including data from incomplete placental material (e.g., placenta previa/accreta) or pathologically enlarged placentas (e.g., hydropic placentas). Umbilical vein and artery PO_2_, PCO_2_, and pH values were “cleaned” with values < or > 3 SD from their respective means reviewed in relation to each patient`s individual values, and corrected if likely to have been mis-entered (e.g., mis-placed decimal point, wrong blood gas/pH category), retained unchanged if extreme but plausible, (e.g., high PCO_2_ values with low PO_2_ and pH values), and excluded if extreme and implausible. Cord gas and pH values were then “validated” using the criteria of Westgate et al. [26], with values excluded when deemed to be unphysiological with the umbilical vein PO_2_ < umbilical artery PO_2_ or umbilical vein PCO_2_ > umbilical artery PCO_2_. When there was a high likelihood the same vessel was sampled twice, this was ascribed to the venous cord sample recognizing the ease of sampling the vein compared with the artery. Remaining umbilical vein and artery PO_2_, PCO_2_, and pH values < 0.5th percentile and > 99.5th percentile were additionally excluded, thereby removing extreme blood gas/pH values more likely to be reflective of pronounced intrapartum events (e.g., fetal asphyxia, maternal hyperventilation), and thereby less reflective of “pre-labor” values.

Umbilical vein and artery O_2_ saturation values were calculated from respective PO_2_ and pH values using the empirical equation of Hellegers et al. [27]. We have confirmed this equation to be highly accurate for values > 20%, but to progressively underestimate measured values between 20 and 10%, with calculated values falling to zero thereafter [28]. Accordingly, this empirical equation was used for calculated values > 20% but corrected using a linear adjustment for calculated values < 20% and > 1%, and with calculated values < 1% arbitrarily set to 5%, recognizing that measured values > 5% will be underestimated and measured values < 5% will be overestimated. Fractional O_2_ extraction values were then calculated from respective umbilical vein and artery O_2_ saturation values as (vein O_2_ saturation − artery O_2_ saturation)/vein O_2_ saturation.

The effect of fetal sex on birth weight, placental weight, birth/placental weight ratio, umbilical vein and artery PO_2_, O_2_ saturation, and fractional O_2_ extraction values was examined, along with the relationship to pregnancy and labor/delivery characteristics as potentially confounding covariates. Data are presented as percentages and means ± SD. Pregnancy, labor and delivery characteristics by fetal sex grouping were compared using analysis of variance with post hoc Dunnett’s test for continuous variables and Chi squared tests with Bonferroni adjustments for categorical variables. Comparisons of birth/placental weight ratio and cord O_2_ findings by fetal sex grouping for all-patients were made using analysis of covariance adjusting for potentially confounding variables, and for low-risk patients were made using analysis of variance with no adjustment for any of the covariates. For all analyses *p* values < 0.05 were deemed statistically significant. The impact of advancing gestational age and maternal diabetes on the primary outcomes has/will be reported separately [29].

## Results

### Characteristics of the study population

There were 69,836 patients meeting the study inclusion criteria of whom 36,598 met the low-risk criteria. The pregnancy and labor/delivery characteristics for all patients by fetal sex grouping are shown in Tables 1 and 2, respectively. The data for the pregnancy, labor and delivery characteristics outlined were available for > 98% of patients, except for maternal BMI which was only available for 69% of patients largely, because pre-pregnant weight and height were not collected in the first 3 years of study.

**Table 1:** Pregnancy characteristics by fetal sex

	Males *N* = 35,749	Females *N* = 34,087
Maternal age (years)	29.0 ± 5.4	29.0 ± 5.3
Parity	0.84 ± 1.04	0.84 ± 1.04
Maternal BMI	24.7 ± 5.5	24.7 ± 5.6
Smoking (%)	10.1	10.1
Chronic hypertension (%)	1.3	1.3
Pregnancy induced hypertension (%)	9.9	9.4*
Gestational diabetes (%)	4.0	3.7
Overt diabetes (%)	0.9	0.9
PPROM (%)	2.3	2.0^p = 0.05^
Gestational age categories		**
Near term delivery (%)	5.1	4.4
Term delivery (%)	76.8	78.1
Post-term delivery (%)	18.1	17.5

Data presented as percentages and means ± SD; *p < 0.05, **p < 0.01, vs respective Male value. *BMI* body mass index, *PPROM* preterm premature rupture of membranes, Near term delivery = 35 0/7 to 36 6/7 weeks, Post-term delivery ≥ 41 weeks

**Table 2:** Birth, labor and delivery characteristics by fetal sex

	Males *N* = 35,749	Females *N* = 34,087
Birth weight (g)	3525 ± 521	3393 ± 490***
Placental weight (g)	685 ± 141	671 ± 138***
Birth weight categories		
SGA (%)	8.4	8.3
AGA (%)	79.6	80.1
LGA (%)	12.0	11.6
Anesthetic use		**
Regional (%)	74.5	73.7
General (%)	2.2	2.0
None/Other (%)	23.3	24.3
Delivery type		**
Vaginal (%)	81.1	82.8
Labor CS (%)	12.8	10.5
Elective CS (%)	6.2	6.7
NRFHR for CS (%)	3.2	2.3**
Umbilical artery pH	7.247 ± 0.063	7.249 ± 0.063**
Umbilical artery pH < 7.1 (%)	2.2	2.1

Data presented as percentages and means ± SD; **p < 0.01, ***p < 0.001 vs respective Male value. *SGA* small for gestational age, *AGA* appropriate for gestational age, *LGA* large for gestational age, *CS* cesarean section, *NRFHR* non-reassuring FHR

### Birth weight, placental weight, and birth to placental weight ratios

Birth weights were available for all but four of the 69,836 patients, while placental weights were available for 94.5% of patients after exclusions and missing data. As expected, birth weights and placental weights were lower in females than males (both *p* < 0.001, Table 2), and notably with relative differences less for placental weights than birth weights. Birth/placental weight ratios were likewise available for 94.5% of patients and are shown by fetal sex grouping for the all-patient and low-risk patient populations in Tables 3 and 4, respectively. For all-patients, birth/placental weight ratios were lower in females than males, 5.18 ± 0.84 vs 5.27 ± 0.85 (*p* < 0.001), indicating that birth weights were decreased in size relative to placental weights and consistent with the lower relative differences noted for placental weights. For the low-risk patients, birth/placental weight ratios similarly were lower in females than males, 5.28 ± 0.82 vs 5.37 ± 0.84 (*p* < 0.001), with these values higher than respective all-patient values, indicating that birth weights were increased in size relative to placental weights in low-risk vs all-patients for both male and female infants.

**Table 3:** Birth/placental weight ratios, umbilical cord blood PO_2_ (mmHg), O_2_ saturation (%) and fractional O_2_ extraction values in all patients

	Males *N* = 35,749	Females *N* = 34,087
Birth/placental weight	5.27 ± 0.85	5.18 ± 0.84***
Umbilical vein PO_2_	27.3 ± 6.5	27.7 ± 6.4***
Umbilical artery PO_2_	15.4 ± 5.4	15.2 ± 5.2***
Umbilical vein O_2_ saturation	60.4 ± 16.1	61.7 ± 15.6***
Umbilical artery O_2_ saturation	25.8 ± 12.8	25.4 ± 12.3***
Fractional O_2_ extraction	0.57 ± 0.17	0.58 ± 0.16***

Data presented as means ± SD; ***p < 0.001 vs respective Male value adjusting for the effect of parity, smoking, chronic and pregnancy-induced hypertension, gestational and overt diabetes, gestational age at delivery, and maternal body mass index for birth/placental weight, and additionally for anesthetic use, delivery type and non-reassuring FHR for cesarean delivery for the O_2_ measurements

**Table 4:** Birth/placental weight ratios, umbilical cord blood PO_2_ (mmHg), O_2_ saturation (%) and fractional O_2_ extraction values in low-risk patients

	Males *N* = 18,335	Females *N* = 18,263
Birth/placental weight	5.37 ± 0.84	5.28 ± 0.82***
Umbilical vein PO_2_	27.9 ± 6.1	28.3 ± 6.1***
Umbilical artery PO_2_	15.9 ± 5.2	15.7 ± 5.1***
Umbilical vein O_2_ saturation	62.4 ± 14.8	63.5 ± 14.5***
Umbilical artery O_2_ saturation	26.8 ± 12.9	26.2 ± 12.4***
Fractional O_2_ extraction	0.57 ± 0.17	0.58 ± 0.17***

Data presented as means ± SD; ***p < 0.001 vs respective Male group unadjusted for any covariates. Low-risk = no smoking, hypertension, diabetes, small or large for gestational age, delivery < 37 weeks, or delivery by cesarean section

### Umbilical cord PO_2_, O_2_ saturation, and fractional O_2_ extraction values

Umbilical vein and artery PO_2_ were available for 95.2% and 86.7% of patients, respectively, after exclusions and missing data. Umbilical cord PO_2_, O_2_ saturation, and fractional O_2_ extraction values are shown by fetal sex grouping for the all-patient and low-risk patient populations in Tables 3 and 4, respectively. Fetal sex impacted cord oxygen values for all-patients, with venous PO_2_ increased in females from that in males, 27.7 ± 6.4 vs 27.3 ± 6.5 mmHg (*p* < 0.001), while arterial PO_2_ was marginally decreased in females from that in males, 15.2 ± 5.2 vs 15.4 ± 5.4 mmHg (*p* < 0.001). In the low-risk patients, venous PO_2_ was similarly increased in females, 28.3 ± 6.1 vs 27.9 ± 6.1 mmHg (*p* < 0.001), while arterial PO_2_ was similarly decreased in females, 15.7 ± 5.1 vs 15.9 ± 5.2 mmHg (*p* < 0.001), from that in males. Notably, all of the low-risk PO_2_ values were higher than respective all-patient values. Changes in O_2_ saturation by fetal sex grouping were similar to that of PO_2_ for both patient populations with venous values increased in females, while arterial values were decreased in females (all *p* < 0.001), from that in males. Accordingly, fractional O_2_ extraction values were increased in females when compared to males, in all-patients at 0.58 ± 0.16 vs 0.57 ± 0.17, and in the low-risk patients at 0.58 ± 0.17 vs 0.57 ± 0.17, (both *p* < 0.001).

## Discussion

In this large population-based study, several pregnancy and labor/delivery characteristics were found to differ in relation to fetal sex. Mothers with male infants had increased pregnancy induced hypertension, preterm premature rupture of membranes, preterm and post-term delivery, as well as gestational diabetes although this was not significant. These mothers also had increased regional and general anesthetic use, laboring cesarean sections, and non-reassuring FHR for cesarean section. These findings are similar to those previously reported and support the conjecture that having a male infant is a risk factor for adverse pregnancy outcomes [23, 24, 30]. Since these characteristic differences could impact birth/placental weight ratio and umbilical cord oxygen values, their confounding effects were adjusted for when determining the effect of fetal sex for the all-patient analysis or limited by also studying a subgroup of low-risk patients.

Birth/placental weight ratios were lower in females than males in both the all-patient and low-risk patient populations indicating a decrease in placental efficiency. These findings are similar to those reported in other large population-based studies [4–7] and could involve sex-related differences in placental oxygen transport as a primary determinant of fetal growth in humans [10]. This transport involves diffusion of dissolved oxygen from the maternal blood plasma of the intervillous space across the syncytiotrophoblast–capillary endothelium to the fetal blood plasma of the villi and will be impacted by utero-placental blood flow, placental barrier surface area/thickness and consumption of oxygen, and the oxygen gradient generated by the positive PO_2_ difference between maternal and fetal blood [2, 11–13]. As such, umbilical vein PO_2_ and O_2_ saturation can be seen as functional measures of placental oxygen transport or delivery. However, these values were increased in females and do not support a decrease in placental nutrient transport for oxygen, as a mechanism underlying their lower birth/placental weight ratios compared to that in males.

The increase in umbilical vein oxygen values in females which was seen in both study populations indicates a sex-related difference in some aspect of placental oxygen transport. Notably, uterine artery Doppler resistance indices as measures of vascular resistance are lower as studied from mid to late pregnancy in women with a female compared to a male fetus [14, 15, 18]. This decrease in uterine vascular resistance has been attributed to associated increases in human chorionic gonadotrophin (HCG) hormone in pregnancies with a female fetus acting through angiogenic/vasodilating mechanisms as an adaptive cardiovascular process from early pregnancy [14, 15]. The resulting increase in uterine blood flow in these pregnancies will increase oxygen delivery to the maternal side of the placenta, PO_2_ in the intervillous space and the PO_2_ gradient across the placenta, thereby increasing umbilical vein oxygen values as presently seen. Gross placental structure studied in low-risk pregnancies shows few sex-related differences; however, measurements relating to lateral chorionic disk expansion are generally less in females suggesting female placentae grow slower earlier in pregnancy [20]. This delayed development might have its basis in higher uterine blood flows and thereby higher intervillous space PO_2_ in females which would then be less of a stimulus for terminal villi growth and vascularization [13] which are also reported to be decreased in female placentae [19].

Umbilical artery oxygen values were instead marginally decreased in females compared to males in both study populations indicating a sex-related decrease in fetal O_2_ delivery relative to consumption as determinants of fetal systemic oxygenation. While these values are directly related to umbilical vein oxygen values, it is both the dissolved and hemoglobin-bound fractions that determine the oxygen content delivered to the fetus and available for uptake from the umbilical circulation [11–13]. Female infants have lower hemoglobin concentrations and a lower proportion of fetal to adult hemoglobin in cord blood at birth (~ 2% on average) [31–33] which will decrease oxygen content thereby decreasing fetal O_2_ delivery compared to males. Umbilical artery resistance indices as measures of vascular resistance on the fetal side of the placenta are higher from mid to late pregnancy in women with a female fetus [16–18], which may relate to a slower growth trajectory of terminal villi and vascularization as determinants of umbilical vascular resistance [13, 19]. The resulting decrease in umbilical blood flow will further the decrease in fetal O_2_ delivery compared to males. While male fetuses have increased total body growth more of this is carcass with a lower metabolic rate, whereas females have internal organs which develop faster and increased percent body fat mass with higher metabolic rates [6, 13, 30, 34–36]. As such, fetal O_2_ consumption/unit body weight may be higher in females which is supported by their higher heart rates and relationship to O_2_ consumption/unit body weight [13, 16, 17, 23] and the finding of increased O_2_ consumption in female newborns compared to males [21]. Overall, these sex-related differences are likely to result in a decrease in fetal O_2_ delivery relative to consumption in females providing a physiologic basis for the decreased umbilical artery oxygen values presently seen.

Fetal O_2_ consumption is equal to the product of umbilical blood flow and veno-arterial O_2_ content difference, while fetal O_2_ delivery is equal to the product of umbilical blood flow and vein O_2_ content [12, 22]. Fetal O_2_ extraction is the ratio between fetal O_2_ consumption and O_2_ delivery (umbilical veno-arterial O_2_ content difference/vein O_2_ content) thereby providing a measure of O_2_ consumption as a fraction of that delivered [12, 22]. Since O_2_ content is directly proportional to the O_2_ saturation of blood, this parameter can instead be used [13, 28]. Fetal O_2_ extraction values were marginally increased in females compared to males in both study populations indicating an increase in O_2_ consumption relative to delivery as expected given the cord oxygen findings. The extent to which fetal O_2_ extraction can increase to maintain O_2_ consumption should O_2_ delivery fall has clinical importance as an indication of the oxygen reserve available to the fetus [12, 22]. SGA infants and those born post-term have higher O_2_ extractions at birth suggesting a lower oxygen reserve for hypoxic-related events [10, 28, 29]. The increased O_2_ extraction and lower systemic oxygen values seen in females while indicating small differences, likewise suggest a lower oxygen reserve for hypoxic-related events. As such other protective strategies must underlie their decreased hypoxic-related morbidity/mortality compared to males [23, 24], including the ability to reduce fetal growth when oxygenation is compromised as suggested by the studies of Clifton et al. [37].

Study limitations include the extent to which labor and delivery affect cord O_2_ findings, with pulse oximetry showing O_2_ saturation to be marginally decreased through labor/delivery [38]. However, cord O_2_ findings at birth will relate to pre-labor/delivery oxygenation and can be reflective of this if the sample size is sufficient and covariates controlled for with large population-based studies as we have reported [10, 28, 39]. Oxygen saturation values were calculated from the umbilical cord PO_2_ and pH data with a previously derived empirical equation and, although highly accurate for values > 20%, will progressively underestimate measured values between 20 and 10%, with calculated values falling to zero thereafter [27, 28]. We, therefore, corrected calculated values < 20% and > 1% using a linear adjustment which should be fairly accurate; however, re-setting calculated values < 1% arbitrarily to 5% which applied to 1.5% of the arterial values, is still likely to underestimate these values on average. Nonetheless, this underestimation should not negate the significant effect of fetal sex on these values which overall were in keeping with those we [10, 28] and others [40] have reported. Finally, placentae were not trimmed, but weighed with membranes and umbilical cord. However, this weighing of placentae was consistently used and any increase in population variance should not bias the internal validity of results; furthermore, a strong correlation (*r* = 0.98) has been reported between trimmed and non-trimmed placentae [41].

## Perspectives and significance

The present findings extend the birth/placental weight ratio narrative [1, 3, 4] now adding umbilical vein and artery O_2_ outcomes and studying a patient population of sufficient size controlling for multiple covariates and including a low-risk group to better delineate the effects of fetal sex. Birth/placental weight ratios were lower in females as previously reported [4–7] and indicating a decrease in placental efficiency compared to males. Umbilical vein O_2_ values were higher in females which could be attributed to HCG-mediated increases in uterine blood flow as an adaptive cardiovascular process from early pregnancy [14, 15, 18]. Umbilical artery O_2_ values were instead lower in females which could be attributed to lower blood oxygen-carrying capacity [31–33] and umbilical blood flow [16–18] and thereby fetal O_2_ delivery relative to O_2_ consumption [13, 21, 34–36] than in males. The increase in umbilical vein O_2_ values in females does not support a decrease in oxygen transport across the placenta as a mechanism underlying their lower birth/placental weight ratios. However, umbilical arterial blood formed by the mixing of umbilical venous oxygenated blood with the venous effluent of fetal tissues is a better measure of systemic oxygenation in the fetus and the oxygen levels signaling fetal growth [10–13, 22]. These values were decreased in females and could account for their lower birth weights compared to males. Moreover, delayed development in female placentae from early pregnancy with the growth trajectory generally slower compared to males [20, 42] could account for their lower placental weights. As such, sex-related differences in placental development as discussed, can be linked to the cord oxygen values observed and the differences in birth weights and placental weights, which could be differentially effected contributing to the birth/placental weight findings we and others [4–7] have noted. Notably the sex-related differences in oxygen values herein reported are small, especially for the umbilical artery. However, small changes in PO_2_ will give rise to larger changes in fetal oxygenation given the slope of the oxyhemoglobin dissociation curve, and arterial values will normalize to the extent that growth is decreased as an oxygen consuming process in response to a lowering in fetal oxygenation [12, 13]. Furthermore, these small oxygen differences are predicted by known sex-related differences in uterine and umbilical blood flows [14–18], blood oxygen-carrying capacity [31–33], placental development [19, 20], and fetal/newborn O_2_ consumption [13, 21, 34–36] which are also comparatively small. Nonetheless, the interdependency of these sex-related differences now including cord oxygen supports their biologic significance and provides insight for regulatory mechanisms underlying fetal–placental growth differences as noted.

## Data Availability

The data sets generated and analyzed during the current study were obtained from the St Joseph’s Health Care Perinatal Database, London, Ontario, Canada. These data sets are not publicly available due to patient privacy issues. However, de-identified data sets with date of birth removed, can be made available from the authors upon reasonable request and with permission of the London Health Sciences Centre and Western University Human Subjects Research Ethics Board, London, Ontario, Canada.
